# Racial/ethnic disparities in curative‐intent treatment for early‐stage non‐small cell lung cancer patients among heterogeneous Black populations: US‐born Black, Afro‐Haitian, West Indian Black, and Hispanic Black

**DOI:** 10.1002/cam4.7449

**Published:** 2024-10-08

**Authors:** Kamaria T. Jacobs, Qinran Liu, Clyde P. Brown, Gilberto Lopes, Paulo S. Pinheiro

**Affiliations:** ^1^ College of Pharmacy and Pharmaceutical Sciences, Institute of Public Health Florida A&M University Tallahassee Florida USA; ^2^ Department of Public Health Sciences University of Miami School of Medicine Miami Florida USA; ^3^ Department of Surveillance and Health Equity Science American Cancer Society Atlanta Georgia USA; ^4^ Sylvester Comprehensive Cancer Center University of Miami Miami Florida USA

**Keywords:** African American, Black Hispanics, Black subgroups, Comorbidities, Curative‐intent treatment, Haitians, Lung cancer, Racial disparities, Stereotactic body radiation therapy, Surgery, Treatment disparities, US‐born Blacks, West Indians

## Abstract

**Background:**

Heterogeneous Black populations encounter significant obstacles in accessing cancer care, yet research on lung cancer treatment disparities remains limited. This study investigates whether the disparity in receiving curative‐intent treatment (curative‐intent surgery and/or stereotactic body radiation therapy [SBRT]) for early‐stage non‐small cell lung cancer (NSCLC) between non‐Hispanic Whites (NHWs) and total Blacks extends to diverse Black populations, including US‐born, Afro‐Haitian, West Indian Black, and Hispanic Black individuals.

**Methods:**

This cross‐sectional study included all Florida cancer registry early‐stage NSCLC cases 2005–2017, linked to individual‐level discharge data containing comorbidity and specific treatment details (surgery and/or SBRT). Multivariable logistic regression assessed the association between race/ethnicity and the receipt of curative‐intent treatment, while accounting for sociodemographic factors (poverty, age, insurance, and smoking status) and clinical variables.

**Results:**

Among 55,655 early‐stage NSCLC patients, 71.1% received curative‐intent treatment: 72.1% NHW and 59.7% Black (non‐Hispanic and Hispanic) individuals. Black patients had 35% lower odds (OR_adj_, 0.65; 95% CI, 0.59–0.70) of receiving curative‐intent treatment compared to NHW patients. ORs varied from 0.57 (95% CI, 0.59–0.70) for Hispanic Black to 0.76 (95% CI, 0.56–1.02) for West Indian Black. Remarkably, Black‐White disparities persisted despite the availability of curative treatment options (SBRT) for both high Charlson Comorbidity Index (CCI) observed among US‐born Blacks and surgery for low CCI patients among all other Black subgroups.

**Conclusions:**

Pronounced disparities in accessing curative‐intent treatments for early‐stage NSCLC were evident across all Black subgroups, regardless of treatment availability and comorbidity profile. These findings underscore the need to address Black heterogeneity and prompt further research to rectify treatment disparities in early‐stage NSCLC.

## 1 INTRODUCTION

Lung cancer is both one of the most common cancers and a leading cause of death worldwide.^1^ In the US, it is the second most common cancer among men and women, with an estimated 238,340 new cases and 127,070 deaths in 2023 alone.^1^ Incidence rates are notably higher among Black Americans in comparison to their White counterparts,^1^, ^2^ and their survival rates are lower.^3^, ^4^ Non‐small cell lung cancer (NSCLC) accounts for approximately 84% of lung cancer cases,^1^ and early‐stage NSCLC is potentially curable^5^ but <30% of these cases are diagnosed at Stage I or II.^1^, ^6^

Long‐standing inequities in treatment of lung cancer among Black and White patients have been well‐documented.^3^, ^4^, ^7^, ^8^, ^9^, ^10^, ^11^, ^12^, ^13^, ^14^ Standard curative‐intent treatment recommendations for early‐stage NSCLC include surgical resection or stereotactic body radiotherapy (SBRT).^5^, ^15^, ^16^ Several studies have reported Black patients are less likely to undergo any curative‐intent surgical treatment.^2^, ^3^, ^4^, ^7^, ^8^, ^9^, ^10^, ^11^, ^12^, ^13^, ^17^ However, SBRT is a more suitable treatment option for early‐stage NSCLC patients who are inoperable or have multiple comorbidities, conditions which are more common among Black Americans.^5^, ^15^, ^16^ Despite this, studies have shown that Black patients (in aggregate) are less likely to undergo curative‐intent SBRT as well.^7^, ^10^, ^14^, ^18^, ^19^

Black populations are highly heterogeneous regarding various determinants of health, including socioeconomic status (SES) and lifestyle behavior in association with country/region of origin and educational levels, insurance coverage, healthcare accessibility, tobacco and alcohol consumption, and dietary patterns.^8^, ^17^, ^20^, ^21^, ^22^, ^23^ These factors likely vary between Black ethnic subgroups and may impact receipt of curative‐intent treatment. This diversity also extends to clinical factors, including genomic variation, tumor characteristics, stage of diagnosis and the prevalence of comorbidities.^24^, ^25^, ^26^, ^27^ Specifically, the proportions of lung cancer in ever and never smokers are substantially different between US‐born, Haiti‐born, and West Indian‐born Blacks,^28^ yet little is known on the characteristics of NSCLC in these populations. Given that smoking is associated with a higher likelihood of comorbidities, it is plausible that distinct disparities exist in terms of receipt of curative‐intent treatment, surgery or SBRT, among Black subgroup populations. Moreover, it remains unclear whether the known treatment disparities impact all Black subgroups uniformly. Florida exemplifies this diversity with sizable populations of US‐born Black, Afro‐Haitian Black, West Indian Black, and Hispanic Black persons.

The aim of this study is to identify differences in the characteristics of early‐stage NSCLC and treatment disparities among Black subgroups. We further assessed if the current documented disparities with NHW are consistent across all Black ethnic subgroups, or if there are specific subgroups of the Black population that require more awareness surrounding treatment disparities. For these purposes, we examined early‐stage NSCLC data from the Florida Cancer Data System (FCDS), the comprehensive statewide cancer registry. Every case documented in the FCDS, complete with demographic, SES, and clinical details, was individually matched with discharge data from the Florida Agency for Health Care Administration (AHCA). This linkage offered unique information on comorbidities and specific curative‐intent treatments (curative‐intent surgery and SBRT) for early‐stage NSCLC, details that are not normally available in standard cancer registries.

## 2 MATERIALS AND METHODS

### 2.1 Data source and study population

FCDS is part of the National Program of Cancer Registries (NPCR), which is administered by the Centers for Disease Control (CDC).^29^ FCDS adheres to the highest standards for timeliness, data quality, and completeness set forth by North American Association of Central Registries (NAACCR), the American College of Surgeons, Commission on Cancer and the Surveillance, Epidemiology and End Results (SEER) reporting program.^29^ Data for FCDS is sourced from 254 hospitals, 108 Radiation Therapy Centers, 515 Surgery Centers and 5688 Physician Offices, ensuring comprehensive coverage across regions including South Florida, Tampa Bay, Central Florida, Northeast Florida, Southwest Florida, and Northwest Rural.^30^

From 2005 to 2017, A total of 2.0% of the initial count of Non‐Hispanic Blacks (NHBs) had to be excluded because of birth in countries others than US, Haiti and West Indies.

A total of 55,993 patients diagnosed with early‐stage NSCLC (AJCC stages I/II and/or SEER localized stage with International Classification of Diseases for Oncology, third edition [ICD‐O‐3] primary site codes C34.X and morphology codes [8001–8035, 8046–9540]) were identified.^31^ Of note, cases diagnosed at autopsy (*N* = 10), patients who died prior to recommended surgery (*N* = 60), patients who died before recommended SBRT (*N* = 120), patients who refused both recommended surgery and radiation therapy (*N* = 148) were excluded from the study (Figure S1).

### 2.2 Treatment and comorbidity identification

The primary outcome of interest was receipt of curative‐intent treatment, defined as either curative‐intent surgery and/or SBRT. Curative‐intent treatment data was obtained from a linkage between FCDS and AHCA from 2005 to 2017, identifying treatments from all available records starting at diagnosis and continuing through the patient's first course of treatment, normally up to 6 months after diagnosis. AHCA provides a population‐based hospital discharge dataset, encompassing diagnoses and procedures for each hospitalization or outpatient encounter in Florida. As detailed in a different study this linkage was performed based on first and last name, gender, age, birth date, social security number, and county of residence.^32^ Overall, 94% of selected cases from FCDS were successfully matched to ACHA data.

Curative‐intent treatment and comorbid conditions were identified from AHCA data based on the International Classification of Diseases, Ninth Revision, Clinical Modification (ICD‐9‐CM), the International Classification of Diseases, Tenth Revision, Clinical Modification/Procedure Coding System (ICD‐10‐CM/PCS), and Current Procedural Terminology/Healthcare Common Procedure Coding System codes (CPT‐4) as detailed in a prior study.^14^ Curative‐intent surgery was defined as any code of sleeve, segmental, and wedge resection, lobectomy, or pneumonectomy. Additionally, any code descriptive of stereotactic radiotherapy (SRS), stereotactic body radiation therapy (SBRT), and stereotactic ablative radiotherapy (SABR) was considered of curative‐intent treatment and is collectively referred to as SBRT for simplicity in this study.^5^ SBRT was defined as thoracic radiotherapy administered at a total radiation dose of 45 Gray or more, delivered in five or fewer fractions, in line with the US billing code standards.^33^

Comorbid conditions were identified from FCDS based on ICD‐9‐CM and ICD‐10‐CM codes at the time of diagnosis for each patient. The comorbidities of each patient were assessed using the modified Charlson Comorbidity Index (CCI), which computes the sum of 15 chronic conditions excluding COPD. Each condition is assigned a weight as previously described.^34^ The CCI for each patient was calculated and categorized into three groups: 0, 1–2, or ≥3 as in previous research.^9^, ^25^ Of note, a CCI score of 0 does not imply the absence of any comorbidities but rather indicates that none of the 16 specific conditions listed in the CCI were present in the patient.^34^

### 2.3 Other covariates

Other studied variables included SES, sex, age at diagnosis, race/ethnicity, neighborhood poverty level (0%–<5% poverty, 5%–<10% poverty, 10%–<20% poverty, 20%–100% poverty, unknown), insurance status (not insured, self‐pay, private, Medicaid, Medicare, others, unknown), region of residence (South Florida, Tampa Bay, Central Florida, Northeast Florida, Southwest Florida, Northwest Rural), and marital status (not married, married, unknown).

For self‐reported race/ethnicity, only non‐Hispanic Whites (NHW) and individuals of Black race regardless of ethnicity were selected for this study. Within the Black race population, the following four distinct ethnic subgroups were identified based on Hispanic ethnicity, Black race, and country of birth: non‐Hispanic US‐born Black, non‐Hispanic Haiti‐born Black (here called Afro‐Haitian), non‐Hispanic West Indies‐born Black, and Hispanic Black (based solely on race and ethnicity and regardless of country of birth). The West Indian countries of birth comprised Jamaica, Trinidad and Tobago as well as all other non‐Hispanic Caribbean Islands and territories as described elsewhere.^35^ A total of 2.0% of the initial count of NHBs had to be excluded because of birth in countries others than US, Haiti and West Indies. Those with missing country of birth accounted for 17.5% of all NHBs and were allocated according to the majority group (US, Haiti or West Indies, and others [excluded]) residing in the same census tract, following methodology in another population‐based study on Florida African descent populations.^36^

Smoking status at diagnosis was categorized as nonsmoker, current smoker, former smoker and unknown. Clinical‐related factors studied included: American Joint Committee on Cancer (AJCC) stage (6th edition of the AJCC staging system from 2005 to 2009; the 7th edition from 2010 onward) (Stage I, Stage II, unknown if stage I/II), histology [adenocarcinoma (8140–8550), squamous cell carcinoma (8050–8084), large cell carcinoma (8012–8013), unspecified and others (8001–8011, 8014–8035, 8123, 8551–9540), NSCLC not otherwise specified (NOS) (8046)], and cancer sequence number (1 primary in the lifetime, 1st of 2 or more primaries, 2nd of 2 or more primaries, ≥3rd of 2 or more primaries).

### 2.4 Statistical analysis

Baseline characteristics were compared between racial/ethnic groups and curative‐intent treatment. The receipt of curative‐intent treatment was classified into four mutually exclusive categories: only curative‐intent surgery, only SBRT, both curative‐intent surgery and SBRT, or not receiving any curative‐intent treatment. Comparative evaluations were conducted using Dunnett's *C*‐test for continuous variables and chi‐square goodness of fit tests for categorical variables.

To understand the depth of disparities in receipt of treatment, associations were assessed between race‐ethnicity and receipt of any type of curative‐intent treatment (surgery and/or SBRT) using multivariable logistic regression. Analyses were first performed between the total Black population and NHW, followed by comparisons between detailed Black ethnic subgroups and NHW. Adjustment for SES, smoking and clinical factors were made in three sequential models: Model 1 adjusted for age; Model 2 adjusted for age and comorbidities; Model 3 adjusted for sociodemographic (age, sex, poverty level, health insurance status, region, and marital status), smoking status, and other clinical factors (diagnosis year, AJCC stage, histology, cancer sequence number, and comorbidities).

Statistical tests with *p* < 0.05 were considered statistically significant. All tests were two‐sided. Data were analyzed with SAS® Statistical Software version 9.4 (SAS Institute Inc., Cary, NC) and Stata version 15.1 (Stata Corp, Texas).

## 3 RESULTS

The total study population consisted of 55,655 patients with early‐stage NSCLC: 51,308 (92.2%) were NHW, 3,‐706 (6.7%) were US‐born Black, 186 (0.3%) were Afro‐Haitian 294 (0.5%) were West Indian Black, and 161 (0.3%) were Hispanic Black. The median age at diagnosis was 71.1 years. While Afro‐Haitian, West Indian Black were by definition foreign‐born, the proportion of foreign‐born among NHWs and Black Hispanics were 5.0% and 77.6% respectively. Females represented 51.3% of the population, slightly surpassing the males at 48.7%. NHW patients had a slightly higher female representation (51.7%), while the majority of Black patients were male (54.2%) (*p* < 0.01) (Table 1).

**TABLE 1:** Characteristics of early‐stage NSCLC population by race/ethnicity, Florida, 2005–2017 (*N* = 55,655).

	*N* (%)							*p‐*value^a^	*p‐*value^b^
	Non‐Hispanic White	Total Black (non‐Hispanic and Hispanic)^c^	US‐born Black	Afro‐Haitians Black	West Indian Black	Hispanic Black	Total		
Total	51,308 (92.2)	4,347 (7.8)	3,706 (6.7)	186 (0.3)	294 (0.5)	161 (0.3)	55,655 (100)		
Mean age at diagnosis (SD)	71.4 (10.1)	67.8 (10.9)	67.6 (10.8)	66.2 (12.5)	69.7 (10.8)	69.6 (11.1)	71.1 (10.2)	<0.01	<0.01
Foreign born (outside US‐50)	2,542 (5.0)^d^	‐^e^, ^f^	0 (0)^e^	186 (100)^e^	294 (100)^e^	120 (77.6)^f^	3,742 (6.7)		
Age group									
<55	2,996 (5.8)	471 (10.8)	399 (10.8)	34 (18.3)	27 (9.2)	11 (6.8)	3,467 (6.2)	<0.01	<0.01
55–64	8,857 (17.3)	1,146 (26.4)	1,020 (27.5)	41 (22.0)	51 (17.4)	34 (21.1)	10,003 (18.0)		
65–74	18,797 (36.6)	1,520 (35.0)	1,283 (34.6)	66 (35.5)	113 (38.4)	58 (36.0)	20,317 (36.5)		
75–84	16,337 (31.8)	971 (22.3)	808 (21.8)	33 (17.7)	84 (28.6)	46 (28.6)	17,308 (31.1)		
≥85	4,321 (8.4)	239 (5.5)	196 (5.3)	12 (6.5)	19 (6.5)	12 (7.5)	4,560 (8.2)		
Sex									
Male	24,767 (48.3)	2,356 (54.2)	2,012 (54.3)	91 (48.9)	162 (55.1)	91 (56.5)	27,123 (48.7)	<0.01	<0.01
Female	26,539 (51.7)	1,990 (45.8)	1,693 (45.7)	95 (51.1)	132 (44.9)	70 (43.5)	28,529 (51.3)		
Smoking status									
Never smoker	5,060 (9.9)	702 (16.2)	495 (13.4)	72 (38.7)	87 (29.6)	48 (29.8)	5,762 (10.4)	<0.01	<0.01
Current smoker	14,742 (28.7)	1,439 (33.1)	1,295 (34.9)	40 (21.5)	61 (20.8)	43 (26.7)	16,181 (29.1)		
Former smoker	22,522 (43.9)	1,436 (33.0)	1261 (34.0)	44 (23.7)	96 (32.7)	35 (21.7)	23,958 (43.1)		
Unknown	8,984 (17.5)	770 (17.7)	655 (17.7)	30 (16.1)	50 (17.0)	35 (21.7)	9,754 (17.5)		
Poverty level									
0%–<5% poverty	6,451 (12.6)	170 (3.9)	131 (3.5)	<10 (2.7)	25 (8.5)	<10 (5.6)	6621 (11.9)	<0.01	<0.01
5%–<10% poverty	16,214 (31.6)	545 (12.5)	430 (11.6)	29 (15.6)	62 (21.1)	24 (14.9)	16,759 (30.1)		
10%–<20% poverty	19,499 (38.0)	1,234 (28.4)	1,018 (27.5)	62 (33.3)	97 (33.0)	57 (35.4)	20,733 (37.3)		
20%–100% poverty	8,739 (17.0)	2,360 (54.3)	2,093 (56.5)	88 (47.3)	109 (37.1)	70 (43.5)	11,099 (19.9)		
Unknown	405 (0.8)	38 (0.9)	34 (0.9)	<10 (1.1)	<10 (0.3)	<10 (0.6)	443 (0.8)		
Health insurance status									
Not insured	360 (0.7)	100 (2.3)	77 (2.1)	10 (5.4)	11 (3.7)	<10 (1.2)	460 (0.8)	<0.01	<0.01
Self‐pay	459 (0.9)	85 (2.0)	68 (1.8)	<10 (3.8)	<10 (1.7)	<10 (3.1)	544 (1.0)		
Private	18,498 (36.1)	1,243 (28.6)	1,042 (28.1)	54 (29.0)	98 (33.3)	49 (30.4)	19,741 (35.5)		
Medicaid	3,252 (6.3)	876 (20.2)	752 (20.3)	43 (23.1)	49 (16.7)	32 (19.9)	4,128 (7.4)		
Medicare	26,128 (50.9)	1,772 (40.8)	1,520 (41.0)	62 (33.3)	124 (42.2)	66 (41.0)	27,900 (50.1)		
Other insurances	1683 (3.3)	159 (3.7)	147 (4.0)	<10 (1.6)	<10 (1.0)	<10 (3.7)	1,842 (3.3)		
Unknown	928 (1.8)	112 (2.6)	100 (2.7)	<10 (3.8)	<10 (1.4)	<10 (0.6)	1,040 (1.9)		
Region									
South Florida	9,077 (17.7)	1,319 (30.3)	913 (24.6)	140 (75.3)	161 (54.8)	105 (65.2)	10,396 (18.7)	<0.01	<0.01
Tampa Bay	10,405 (20.3)	710 (16.3)	661 (17.8)	14 (7.5)	13 (4.4)	22 (13.7)	11,115 (20.0)		
Central Florida	11,797 (23.0)	730 (16.8)	641 (17.3)	18 (9.7)	59 (20.1)	12 (7.5)	12,527 (22.5)		
Northeast Florida	8,745 (17.0)	910 (20.9)	856 (23.1)	<10 (3.2)	35 (11.9)	13 (8.1)	9,655 (17.4)		
Southwest Florida	7,749 (15.1)	251 (5.8)	219 (5.9)	<10 (3.2)	19 (6.5)	<10 (4.4)	8,000 (14.4)		
Northwest Rural	3,535 (6.9)	427 (9.8)	416 (4.0)	<10 (1.6)	<10 (1.0)	<10 (3.7)	3,962 (3.3)		
Marital status									
Not married	21,549 (42.0)	2,587 (59.5)	2,265 (61.1)	96 (51.6)	142 (48.3)	84 (52.2)	24,136 (43.4)	<0.01	<0.01
Married	28,530 (55.6)	1,609 (37.0)	1,310 (35.4)	82 (44.1)	146 (49.7)	71 (44.1)	30,139 (54.2)		
Unknown	1,229 (2.4)	151 (3.5)	131 (3.5)	<10 (4.3)	<10 (2.0)	<10 (3.7)	1,380 (2.5)		
Diagnosis year									
2005–2009	17,628 (34.4)	1,357 (31.2)	1,153 (31.1)	56 (30.1)	89 (30.3)	59 (36.7)	18,985 (34.1)	<0.01	<0.01
2010–2014	18,541 (36.1)	1,615 (37.2)	1,378 (37.2)	66 (35.5)	117 (39.8)	54 (33.5)	20,156 (36.2)		
2015–2017	15,139 (29.5)	1,375 (31.6)	1,175 (31.7)	64 (34.4)	88 (29.9)	48 (29.8)	16,514 (29.7)		
AJCC cancer stage									
Stage I	36,770 (71.7)	2,883 (66.3)	2433 (65.7)	133 (71.5)	198 (67.4)	119 (73.9)	39,653 (71.3)	<0.01	<0.01
Stage II	8,844 (17.2)	835 (19.2)	726 (19.6)	26 (14.0)	55 (18.7)	28 (17.4)	9,679 (17.4)		
Unknown if stage I/II	5,694 (11.1)	629 (14.5)	547 (14.8)	27 (14.5)	41 (14.0)	14 (8.7)	6,323 (11.4)		
Histology									
Adenocarcinoma	27,412 (54.0)	2,262 (52.8)	1,854 (50.7)	126 (70.0)	185 (64.9)	97 (61.0)	29,674 (53.9)	<0.01	<0.01
Squamous cell carcinoma	14,763 (29.1)	1,217 (28.4)	1,107 (30.3)	23 (12.8)	54 (19.0)	33 (20.8)	15,980 (29.0)		
Large cell carcinoma	990 (2.0)	92 (2.2)	79 (2.2)	<10 (1.1)	<10 (2.1)	<10 (3.1)	1,082 (2.0)		
Unspecified and Others	3,241 (6.4)	307 (7.2)	263 (7.2)	18 (10.0)	16 (5.6)	10 (6.3)	3,548 (6.4)		
NSCLC NOS	4,381 (8.6)	403 (9.4)	354 (9.7)	11 (6.1)	24 (8.4)	14 (8.8)	4,784 (8.7)		
Cancer sequence number									
1 primary in the lifetime	29,546 (57.6)	2,792 (64.2)	2,386 (64.4)	125 (67.2)	188 (64.0)	93 (57.8)	32,338 (58.1)	<0.01	<0.01
1st of 2 or more primaries	5,937 (11.6)	504 (11.6)	426 (11.5)	20 (10.8)	31 (10.5)	27 (16.8)	6,441 (11.6)		
2nd of 2 or more primaries	11,788 (23.0)	835 (19.2)	708 (19.1)	35 (18.8)	64 (21.8)	28 (17.4)	12,623 (22.7)		
≥3rd of 2 or more primaries	4,037 (7.9)	216 (5.0)	186 (5.0)	<10 (3.2)	11 (3.7)	13 (8.1)	4,253 (7.6)		
Charlson Comorbidity Index (CCI)									
CCI = 0	13,209 (27.5)	987 (24.3)	803 (23.1)	55 (34.6)	75 (27.9)	54 (35.1)	14,196 (27.3)	<0.01	<0.01
1 ≤ CCI ≤2	28,828 (60.1)	2318 (57.1)	1,991 (57.3)	86 (54.1)	155 (57.6)	86 (55.8)	31,146 (59.8)		
CCI ≥3	5,966 (12.4)	752 (18.5)	681 (19.6)	18 (11.3)	39 (14.5)	14 (9.1)	6,718 (12.9)		
Curative‐intent surgery^g^									
Yes	33,569 (65.4)	2,390 (55.0)	1,995 (53.8)	115 (61.8)	185 (62.9)	95 (59.0)	35,959 (64.6)	<0.01	<0.01
No	17,739 (34.6)	1,957 (45.0)	1,711 (46.2)	71 (38.2)	109 (37.1)	66 (41.0)	19,696 (35.4)		
SBRT^h^									
Yes	6,278 (12.2)	378 (8.7)	334 (9.0)	<10 (4.8)	25 (8.5)	10 (6.2)	6,656 (12.0)	<0.01	<0.01
No	45,030 (87.8)	3,969 (91.3)	3,372 (91.0)	177 (95.2)	269 (91.5)	151 (93.8)	48,999 (88.0)		
Curative‐intent treatment^i^									
Yes	36,973 (72.1)	2,593 (59.7)	2,181 (58.9)	118 (63.4)	194 (66.0)	100 (62.1)	39,566 (71.1)	<0.01	<0.01
No	14,335 (27.9)	1,754 (40.4)	1,525 (41.2)	68 (36.6)	100 (34.0)	61 (37.9)	16,089 (28.9)		

Abbreviations: AJCC, American Joint Committee on Cancer; CCI, Charlson Comorbidity Index; NOS, not otherwise specified; SD, standard deviation; SBRT, stereotactic body radiation therapy.

^a^ *p*‐values obtained from the chi‐square goodness of fit test for White and total Black patients.

^b^ *p*‐values obtained from the chi‐square goodness of fit test for Black subgroups.

^c^ The total number of Black patients is the sum of all patients from Black ethnicity groups.

^d^ 8,372 had unknown birthplace.

^e^ For non‐Hispanic Black groups as the 17.5% with missing birthplace were allocated to the majority group by census tract (see methods).

^f^ 5 had unknown birthplace.

^g^ Curative‐intent surgery (*N* = 35,959) included 32,910 patients received only curative‐intent surgery, and 3049 patients received both curative‐intent surgery and SBRT at the same time.

^h^ SBRT (*N* = 6,656) included 3607 patients received only SBRT, and 3049 patients received both curative‐intent surgery and SBRT at the same time.

^i^ Curative‐intent treatment was defined as receiving specific curative‐intent surgery and/or SBRT.

Afro‐Haitian had the highest proportion of never smokers at 38.7%, followed by Hispanic Black (29.8%) and West Indian Black patients (29.6%). In contrast, US‐born Blacks (13.4%) and White patients (9.9%) had a relatively low proportion of never smokers. Proportions of patients residing in areas with the highest poverty level (20%–100% poverty) were significantly different between NHW (17.0%) and total Black patients (54.3%) (*p* < 0.01). Among Black populations, the proportions varied between 56.5% among US‐born Black patients and 37.1% West Indian Black patients. Regarding Medicaid coverage, the proportions were low for NHW (6.3%) but high for Black patients (20.2%): US‐born Black (20.3%) and Afro‐Haitian Black (23.1%), West Indian Black (16.7%) and Hispanic Black (19.9%). For the uninsured, the most extreme figures were 5.4% for Afro‐Haitian Blacks, and 0.7% for NHW.

The proportion of adenocarcinomas in majority non‐US‐born Black populations (Afro‐Haitian, West Indian Black and Black Hispanic groups) was significantly higher compared to both US‐born Blacks and NHW. This aligns with the understanding that adenocarcinomas are the most common morphology among never smokers, as observed in our findings. Regarding comorbidities, the proportion of patients with a CCI score of 0 (CCI = 0) was lower among total Black patients (24.3%) compared to NHW patients (27.5%) (*p* < 0.01). By Black subgroups this proportion varied significantly: 35.1% among Hispanic Black patients, 34.6% in Afro‐Haitian patients, 27.9% of West Indian Black patients, and 23.1% of US‐born Black patients had CCI = 0 (*p* < 0.01). The majority of patients (59.8%) had a CCI score between 1 and 2 (1 ≤ CCI≤2) among all populations. However, a higher proportion of Black patients (18.5%) had a CCI score of 3 or higher (CCI≥3), compared to NHW patients (12.4%) (*p* < 0.01). This proportion also varied widely among Black ethnic groups: 19.6% of US‐Born Blacks, 14.5% of West Indian Blacks, 11.3% of Afro‐Haitians, and 9.1% of Hispanic Black patients had CCI ≥3.

Of all patients, 64.6% of patients underwent surgery with curative intent: 65.4% of NHW and 55.0% of total Black patients (*p* < 0.01). Among Black subgroups, this proportion varied: 62.9% among West Indian Blacks, 61.8% of Afro‐Haitians, 59.0% of Hispanic Black, and 53.8% of US‐born Black patients (*p* < 0.01). Only 8.7% of total Black patients received SBRT, compared to 12.2% of NHW patients (*p* < 0.01). In terms of SBRT, Afro‐Haitians showed the lowest percentage (4.8%), followed by Hispanic Black (6.2%), West Indian Black (8.5%), and US‐born Black patients (9.0%), although the differences between Black subgroups were not significant (*p* = 0.16). When combining curative‐intent surgery and SBRT into a broader curative‐intent treatment category, NHW patients led with 72.1%, whereas total Black patients lagged at 59.7% (*p* < 0.01). Within Black ethnic subgroups, US‐born Blacks had the lowest proportion at 58.9%, with West Indian‐born Blacks having the highest at 66.0% (*p* = 0.06).

Table 2 shows patient characteristics by curative‐intent treatment groups. Patients who underwent curative‐intent surgery were typically younger, with an average age of 69.3, compared to 74.4 for SBRT only, 74.1 for both treatments, and 73.3 for no treatment (*p* < 0.01). Overall, 59.1% of patients received curative‐intent surgery only, 6.5% received SBRT only, 5.5% received both surgery and SBRT, and 28.9% of patients did not receive any curative‐intent treatment. When comparing racial groups, NHW patients showed a distribution of 59.8% (surgery only), 6.6% (SBRT only), 5.6% (both), and 27.9% (none), while total Black patients displayed a different distribution: 51.0%, 4.7%, 4.0%, and 40.4%, respectively. All Black subgroups had lower proportions of receiving curative‐intent treatment (surgery and/or SBRT) compared to NHW (*p* < 0.05).

**TABLE 2:** Characteristics of study population by curative‐intent treatment groups, Florida, 2005–2017 (*N* = 55,655).

	*N* (%)					*p‐*value^a^
	Curative‐intent surgery only	Curative‐intent SBRT only	Received both curative‐intent surgery and SBRT	No curative‐intent treatment	Total	
Overall	32,910 (59.1)	3,607 (6.5)	3,049 (5.5)	16,089 (28.9)	55,655 (100)	
Mean age at diagnosis (SD)	69.3 (9.7)	74.4 (9.3)	74.1 (9.7)	73,3 (10.6)	71.1 (10.2)	<0.01
Age group						
<55	2,466 (71.1)	88 (2.5)	119 (3.4)	794 (22.9)	3,467 (100)	<0.01
55–64	6,713 (67.1)	455 (4.6)	362 (3.6)	2,473 (24.7)	10,003 (100)	
65–74	13,154 (64.7)	1,183 (5.8)	987 (4.9)	4,993 (24.6)	20,317 (100)	
75–84	9,372 (54.2)	1,340 (7.7)	1,144 (6.6)	5,452 (31.5)	17,308 (100)	
≥85	1,205 (26.4)	541 (11.9)	437 (9.6)	2,377 (52.1)	4,560 (100)	
Race/ethnicity						
Non‐Hispanic White	30,695 (59.8)	3,404 (6.6)	2,874 (5.6)	14,335 (27.9)	51,308 (100)	<0.01^c^
Total Black^b^	2,215 (51.0)	203 (4.7)	175 (4.0)	1,754 (40.4)	4,347 (100)	<0.01^d^
US‐born Black	1,847 (49.8)	186 (5.0)	148 (4.0)	1,525 (41.2)	3,706 (100)	
Afro‐Haitians Black	109 (58.6)	<10 (1.6)	<10 (3.2)	68 (36.6)	186 (100)	
West Indian Black	169 (57.5)	<10 (3.1)	16 (5.4)	100 (34.0)	294 (100)	
Hispanic Black	90 (55.9)	<10 (3.1)	<10 (3.1)	61 (37.9)	161 (100)	
Sex						
Male	15,327 (56.5)	1,828 (6.7)	1,442 (5.3)	8,526 (31.4)	27,123 (100)	<0.01
Female	17,580 (61.6)	1,779 (6.2)	1,607 (5.6)	7,563 (26.5)	28,529 (100)	
Smoking status						
Never smoker	3,665 (63.6)	234 (4.1)	268 (4.7)	1,595 (27.7)	5,762 (100)	<0.01
Current smoker	9,425 (58.3)	1,032 (6.4)	773 (4.8)	4,951 (30.6)	16,181 (100)	
Former smoker	14,262 (59.5)	1,812 (7.6)	1,486 (6.2)	6,398 (26.7)	23,958 (100)	
Unknown	5,558 (57.0)	529 (5.4)	522 (5.4)	3,145 (32.2)	9,754 (100)	
Poverty level						
0%–<5% poverty	4,269 (64.5)	436 (6.6)	369 (5.6)	1,547 (23.4)	6,621 (100)	<0.01
5%–<10% poverty	10,428 (62.2)	1,091 (6.5)	900 (5.4)	4,340 (25.9)	16,759 (100)	
10%–<20% poverty	12,060 (58.2)	1,354 (6.5)	1,137 (5.5)	6,182 (29.8)	20,733 (100)	
20%–100% poverty	5,880 (53.0)	697 (6.3)	633 (5.7)	3,889 (35.0)	11,099 (100)	
Unknown	273 (61.6)	29 (6.6)	10 (2.3)	131 (29.6)	443 (100)	
Health insurance status						
Not insured	253 (55.0)	16 (3.5)	13 (2.8)	178 (38.7)	460 (100)	<0.01
Self‐pay	293 (53.9)	18 (3.3)	11 (2.0)	222 (40.8)	544 (100)	
Private	12,763 (64.7)	1,228 (6.2)	1,120 (5.7)	4,630 (23.5)	19,741 (100)	
Medicaid	1,978 (47.9)	268 (6.5)	198 (4.8)	1,684 (40.8)	4,128 (100)	
Medicare	16,244 (58.2)	1,718 (6.2)	1,587 (5.7)	8,351 (29.9)	27,900 (100)	
Other insurances	906 (49.2)	299 (16.2)	73 (4.0)	564 (30.6)	1,842 (100)	
Unknown	473 (45.5)	60 (5.8)	47 (4.5)	460 (44.2)	1,040 (100)	
Region						
South Florida	6,573 (63.2)	714 (6.9)	586 (5.6)	2,523 (24.3)	10,396 (100)	<0.01
Tampa Bay	6,713 (60.4)	524 (4.7)	588 (5.3)	3,290 (29.6)	11,115 (100)	
Central Florida	7,373 (58.9)	755 (6.0)	634 (5.1)	3,765 (30.1)	12,527 (100)	
Northeast Florida	5,344 (55.4)	763 (7.9)	481 (5.0)	3,067 (31.8)	9,655 (100)	
Southwest Florida	4,820 (60.3)	562 (7.0)	484 (6.1)	2,134 (26.7)	8,000 (100)	
Northwest Rural	2,087 (52.7)	289 (7.3)	276 (7.0)	1,310 (33.1)	3,962 (100)	
Marital status						
Not married	12,938 (53.6)	1,635 (6.8)	1,320 (5.5)	8,243 (34.2)	24,136 (100)	<0.01
Married	19,267 (63.9)	1,857 (6.2)	1,651 (5.5)	7,364 (24.4)	30,139 (100)	
Unknown	705 (51.1)	115 (8.3)	78 (5.7)	482 (34.9)	1,380 (100)	
Diagnosis year						
2005–2009	11,845 (62.4)	834 (4.4)	300 (1.6)	6,006 (31.6)	18,985 (100)	
2010–2014	12,545 (62.2)	1,330 (6.6)	1,312 (6.5)	4,969 (24.7)	20,156 (100)	
2015–2017	8,520 (51.6)	1,443 (8.7)	1,437 (8.7)	5,114 (31.0)	16,514 (100)	
AJCC stage SEER						
Stage I	24,881 (62.8)	2,952 (7.4)	2,060 (5.2)	9,760 (24.6)	39,653 (100)	<0.01
Stage II	5,390 (55.7)	334 (3.5)	479 (5.0)	3,476 (35.9)	9,679 (100)	
Unknown if stage I/II	2,639 (41.7)	321 (5.1)	510 (8.1)	2,853 (45.1)	6,323 (100)	
Histology						
Adenocarcinoma	20,997 (70.8)	1,512 (5.1)	1,623 (5.5)	5,542 (18.7)	29,674 (100)	<0.01
Squamous cell carcinoma	8710 (54.5)	1049 (6.6)	1,068 (6.7)	5153 (32.3)	15,980 (100)	
Large cell carcinoma	687 (63.5)	26 (2.4)	33 (3.1)	336 (31.1)	1082 (100)	
Unspecified and others	246 (6.9)	464 (13.1)	63 (1.8)	2775 (78.2)	3,548 (100)	
NSCLC NOS	1,888 (39.5)	542 (11.3)	235 (4.9)	2119 (44.3)	4,784 (100)	
Cancer sequence number						
1 primary in the lifetime	18,296 (56.6)	1,952 (6.0)	1,424 (4.4)	10,666 (33.0)	32,338 (100)	<0.01
1st of 2 or more primaries	4663 (72.4)	319 (5.0)	374 (5.8)	1,085 (16.9)	6,441 (100)	
2nd of 2 or more primaries	7,439 (58.9)	962 (7.6)	864 (6.8)	3358 (26.6)	12,623 (100)	
≥3rd of 2 or more primaries	2512 (59.1)	374 (8.8)	387 (9.1)	980 (23.0)	4,253 (100)	
Charlson Comorbidity Index (CCI)						
CCI = 0	9,601 (67.6)	648 (4.6)	888 (6.3)	3,059 (21.6)	14,196 (100)	<0.01
1 ≤ CCI ≤2	19,135 (61.4)	1,673 (5.4)	1,666 (5.4)	8,672 (27.8)	31,146 (100)	
CCI ≥3	3,355 (49.9)	493 (7.3)	491 (7.3)	2,379 (35.4)	6,718 (100)	

Abbreviations: AJCC, American Joint Committee on Cancer; CCI, Charlson Comorbidity Index; NOS: not otherwise specified; SD, standard deviation; SBRT, stereotactic body radiation therapy.

^a^ *p*‐value obtained from chi‐squared test.

^b^ The total number of Black patients is the sum of all patients from Black ethnicity groups.

^c^ *p*‐values obtained from the chi‐squared goodness of fit test for White and total Black patients.

^d^ *p*‐values obtained from the chi‐square goodness of fit test for Black subgroups.

Patients who underwent curative‐intent surgery were typically younger, with an average age of 69.3, compared to 74.4 for SBRT only, 74.1 for both treatments, and 73.3 for no treatment (*p* < 0.01). Patients with CCI = 0 (67.6%) had a higher proportion of receipt of curative‐intent surgery only. Individuals with CCI ≥3, had a higher proportion of patients who received both surgery and SBRT (7.3%), or no curative‐intent treatment (35.4%) (Table 2).

Results for the association between race/ethnicity and receipt of curative‐intent treatment are shown in Table 3. Overall, total Black patients had significantly lower odds of receiving any curative‐intent treatment compared to their NHW counterparts, as shown in both the age‐adjusted model (OR: 0.52; 95% CI: 0.49–0.55) and the full multivariable adjusted model (OR: 0.65; 95% CI: 0.59–0.70). In the univariable age‐adjusted model (Model 1), all black ethnic subgroups had significantly lower odds of receiving curative‐intent treatment compared to NHW patients. Specifically, US‐born Blacks had 50% (OR, 0.50; 95% CI, 0.47 to 0.54), Afro‐Haitian Black had 40% (OR, 0.60; 95% CI, 0.45–0.82), Hispanic Black had 39% (OR, 0.61; 95% CI, 0.44–0.84), and West Indian Blacks had 29% (OR, 0.71; 95% CI, 0.56–0.91) lower odds to receive such treatment compared to NHW patients, respectively. After further adjusting for comorbidities (Model 2), the significance for West Indian Blacks (OR, 0.79; 95% CI 0.61–1.03; *p* = 0.07) was no longer present, while the results for the rest of the black ethnic subgroups remained similar to those in the age‐adjusted model.

**TABLE 3:** Determinants of receipt of any curative‐intent treatment (surgery and/or SBRT), Florida, 2005–2017 (*N* = 55,655).

	Odds ratio (95% CI)		
	Curative‐intent treatment (surgery and/or SBRT) (*N* = 39,566) vs. No curative‐intent treatment (*N* = 16,089)		
	Model 1^a^	Model 2^b^	Model 3^c^
Race/ethnicity			
White	1 [Reference]	1 [Reference]	1 [Reference]
Total Black^d^	0.52 (0.49–0.55)	0.52 (0.49–0.56)	0.65 (0.59–0.70)
US‐born Black	0.50 (0.47–0.54)	0.50 (0.47–0.54)	0.64 (0.59–0.69)
Afro‐Haitians Black	0.60 (0.45–0.82)	0.57 (0.41–0.79)	0.61 (0.41–0.89)
West Indian Black	0.71 (0.56–0.91)	0.79 (0.61–1.03)	0.76 (0.56–1.02)
Hispanic Black	0.61 (0.44–0.84)	0.58 (0.41–0.81)	0.57 (0.39–0.82)

Abbreviations: AJCC, American Joint Committee on Cancer; SBRT, stereotactic body radiation therapy.

^a^ Model was adjusted for age.

^b^ Model was adjusted for age and comorbidities.

^c^ Model was adjusted for sociodemographic (age, sex, poverty level, health insurance status, region, and marital status), smoking status, and clinical factors (diagnosis year, AJCC stage, histology, cancer sequence number and comorbidities).

^d^ ORs for total Black were obtained from the model combined all patients from Black ethnicity groups.

With the full adjustment of SES, smoking status, and clinical variables in Model 3, the OR of 0.64 (95% CI: 0.59–0.69) for US‐born Black patients undergoing curative‐intent treatment in comparison to NHW patients showed a moderate attenuation from 0.50 in Model 2 (95% CI: 0.47–0.54), whereas ORs for Afro‐Haitian Black and Hispanic Black patients remained largely unchanged. Females had 21% higher odds of receiving curative‐intent treatment compared to males (OR, 1.21; 95% CI, 1.15–1.26). Compared to those with CCI = 0, individuals with CCI ≥3 and those with 1 ≤ CCI ≤2 had 32% lower odds (OR: 0.68, 95% CI: 0.63–0.73) and 25% lower odds (OR: 0.85, 95% CI: 0.80–0.89), respectively, of receiving curative‐intent treatment (Table S1).

## 4 DISCUSSION

This comprehensive study examines the heterogeneity among distinct Black subgroups concerning early‐stage NSCLC characteristics and their receipt of curative‐intent treatment. We specifically contrasted these subgroups with NHW to emphasize the disparities that persistently affect populations of African descent. Despite the increasing use of SBRT, which could potentially benefit the Black population with a known higher prevalence of comorbidities, we found that even after accounting for comorbidities, SES (insurance status and poverty levels), and smoking status, all Black populations consistently have disadvantages in receipt of curative‐intent treatment for early‐stage NSCLC. This trend aligns with prior research that considered the Black group as an aggregate,^2^, ^3^, ^4^, ^7^, ^8^, ^9^, ^10^, ^11^, ^12^, ^13^, ^14^, ^17^, ^18^, ^19^ indicating that disparities in treatment persist regardless of the treatment modality and comorbidity profile.

Despite the lower receipt of curative intent treatment observed for all four Black subgroups, our analysis revealed two very different profiles within the Black population. The first profile comprises US‐born Blacks and is characterized by higher CCI scores and a greater proportion of smokers, who might potentially benefit more from SBRT. Conversely, the second profile includes Black individuals born in Haiti, the West Indies, or those of Hispanic Black ethnicity. These three groups, overwhelmingly foreign‐born, display substantially lower CCI scores and a lower proportion of ever smokers compared to both NHW and US‐born Black patients, suggesting they could benefit significantly from curative‐intent surgery.^13^, ^25^ This profile aligns with the “Healthy Immigrant Effect” commonly observed in immigrant populations,^37^ which is characterized by the advantages often seen in foreign‐born individuals: a healthier status, evidenced by a lower prevalence of comorbidities and the lower prevalence of smoking among foreign‐born Black populations.^38^ Despite the appropriateness of one or another type of curative treatment for each of these two broad groups within the Black population, namely US‐born and the majority foreign‐born Black groups, they both consistently exhibit reduced odds of receiving both curative‐intent surgery and SBRT compared to NHW patients.

In regard to smoking, the higher proportion of never smokers among Black patients compared to NHW suggests that other environmental risk factors may play a role in the risk of developing NSCLC. This finding is consistent with a previous population‐based study by Pinheiro et al., which reported that the proportion of lung cancer among never smokers (LCNS) was higher in foreign‐born Black populations, such as Haitians and West Indians, compared to White and US‐born populations further highlighting the importance of considering the heterogeneity within Black subgroups when examining risk factors for NSCLC.^28^

Other known social determinants of health impact receipt of treatment by Black populations in our study.^8^, ^17^, ^20^, ^21^, ^22^, ^23^ The association between lower SES and less surgery and less SBRT utilization remains evident. Our data also reveals that Black patients consistently lag in terms of insurance coverage, with many being uninsured or reliant on Medicaid. However, even after adjusting for insurance and SES, the odds of Black populations receiving curative‐intent treatment remained lower than NHWs. This suggests that unaccounted factors in our model, possibly those rooted in systemic racism and discrimination,^39^, ^40^ might still be influencing these observed disparities across all Black groups. Previous studies have laid the landscape in which Blacks are not getting recommended treatment because of the healthcare system's mistrust, clinical care bias, cultural incompetency, language barriers, and patient refusal.^11^, ^13^, ^41^, ^42^ While our analysis excluded patients who refused treatment, it is important to underscore the potential impact of ineffective patient‐physician communication. Lueng et al., reported that language and communication difficulties, limited health literacy, and social support challenges hinder immigrants' navigation of the healthcare system, contributing to disparities.^43^ Black subgroups are culturally and linguistically diverse, with little research supporting significant associations among treatment modalities. Moreover, cultural competency in healthcare professionals targeting patients' racial, ethnic, cultural, and socioeconomic backgrounds can enhance optimal treatment. Socioeconomic status and trust score significantly impact overall communication in NSCLC treatment.^26^ Evans et al. found that optimal treatment modalities partly depend on patients' trust in their physicians.^44^ Previous studies show that obtaining curative surgery can depend on a trusting physician‐patient relationship. Coughlin, Gordon et al. found Blacks, compared to Whites, judged physician‐patient communication as poorly informed and lacking support and partnership.^8^, ^45^ Additionally, Blacks' poor trust in physicians and misconceptions about cancer biology cause many to defer treatment when offered.^44^ Improving physician‐patient communication is crucial for optimal treatment, leading to higher adherence to medical regimens, better decision‐making, and satisfaction in the relationship.^46^, ^47^ Overall, each of these factors may play a different role in the heterogeneous Black subgroups under analysis.^35^ Specifically, Haitian Blacks may face language barriers,^48^ while both Haitians and West Indians may have unique health beliefs,^48^ and US‐born Black patients may experience heightened healthcare mistrust.^42^

The main strength of this study lies in leveraging comprehensive, population‐based cancer registry data, which significantly enhances the generalizability of its findings. This approach surpasses the limitations of typical hospital‐based data, as it avoids convenient sampling and biases related to hospital access or healthcare availability. Another strength arises from Florida's diverse population, allowing for an in‐depth examination of an often‐overlooked population: foreign‐born Black patients from diverse backgrounds. Moreover, the integration of individually linked data on comorbid conditions and specific treatment options adds an important layer of detail to the study, which is not commonly included in population‐based studies which often study surgery and radiotherapy in broad unspecified categories. While many studies have highlighted disparities grounded in income, SES, and geographic location, fewer have provided insights regarding associated comorbidities.^3^, ^9^, ^25^

In regard to generalizability, this study focuses solely on Florida but includes all patients from these specific populations with early‐stage NSCLC. Although the numbers are relatively small, they represent the largest possible cohort within a single state, with the only comparable exception being New York State. The absolute number of African‐descent populations with NSCLC will inevitably increase as the immigrant population ages, making studies like this crucial for highlighting the growing heterogeneity in cancer care among Black populations.

However, it is essential to acknowledge the limitations of this study. First, the demographics of the Black subgroups are confined to Florida, potentially limiting the generalizability to these subgroups in other states. Additionally, the varying population sizes necessitate caution when interpreting the results. Second, the study does not account for other influential variables not collected by cancer registries, including access to the different treatment modalities, treatment compliance, familial support, lifestyle behaviors, and qualitative data on thoughts, feelings, and perceived stigma, all of which might influence treatment decisions. Thirdly, it is possible that certain Black groups, specifically Hispanic Black patients, may not be fully ascertained in cancer registry data, as has been shown in the literature.^49^ Lastly, while the fully adjusted result for West Indian Blacks is not statistically different from one, the low *p*‐value suggests that with a larger population of West Indians and a more precise estimate as a result, the pattern for West Indian Blacks would follow that of other Black subgroups, with lower odds of receipt of curative‐intent treatment compared to NHWs. Furthermore, no causal inferences can be derived from our analysis given the cross‐sectional study design.

To address racial disparities in receiving curative‐intent treatment for NSCLC, stakeholders should implement targeted strategies. Ensuring culturally sensitive communication, providing timely treatment choices, and addressing implicit bias among healthcare providers can build trust and reduce disparities. Language services, particularly for Creole‐speaking Haitian Black populations, can bridge communication gaps. Acknowledging the heterogeneity in health knowledge among Black subgroups is essential for developing effective educational interventions.

In conclusion, this study highlights the critical need to recognize and understand the heterogeneous nature of Black subgroups regarding NSCLC treatment. The uniformly unfavorable receipt of curative‐intent treatment across all Black subgroups calls for action to ensure equitable access to advanced treatment, devoid of biases rooted in race, socioeconomic factors, or location. An in‐depth exploration of structural racism, medical mistrust, and cultural sensitivity within healthcare delivery is necessary. Tailored interventions that acknowledge and address these nuances could play a crucial role in reducing existing disparities. Future research should pivot towards these areas to ensure the development of culturally relevant interventions and solutions.

## AUTHOR CONTRIBUTIONS

**Kamaria T. Jacobs:** Formal analysis (supporting); investigation (equal); software (supporting); writing – original draft (lead); writing – review and editing (equal). **Qinran Liu:** Data curation (equal); formal analysis (lead); investigation (lead); methodology (equal); project administration (equal); software (lead); supervision (equal); validation (equal); writing – review and editing (lead). **Clyde P. Brown:** Conceptualization (equal); funding acquisition (equal); investigation (equal); resources (equal); supervision (equal); validation (equal); writing – review and editing (equal). **Gilberto Lopes:** Writing – review and editing (equal). **Paulo S. Pinheiro:** Conceptualization (equal); data curation (equal); funding acquisition (equal); investigation (equal); methodology (equal); project administration (equal); resources (equal); software (equal); supervision (equal); validation (equal); writing – review and editing (equal).

## FUNDING INFORMATION

This work was supported by grant 20B16 from the Bankhead‐Coley Cancer Biomedical Research Program of the State of Florida (PI Paulo S. Pinheiro).

## CONFLICT OF INTEREST STATEMENT

The authors have no conflicts to disclose.

## ETHICS STATEMENT

This study was approved by the Department of Health of the State of Florida Institutional Review Board (Protocol 2020‐081).

## PATIENT CONSENT STATEMENT

This study was granted exempt status. The research was conducted in compliance with all applicable DOH institutional and IRB policies and procedures, as well as the protections and ethical standards outlined in the US Federal Policy for the Protection of Human Subjects.

## PRECIS

Diverse Black subgroups in Florida, including US‐born Blacks, Afro‐Haitians, and Hispanic Blacks, exhibit significant disparities in receiving curative‐intent treatments for early‐stage NSCLC compared to Whites, despite distinct comorbidity profiles. This underscores the urgent need for research into equitable treatment provision to address these disparities and promote cancer equity.

## Supporting information


Data S1.

Data S2.

## Data Availability

The data generated in this study is available upon request from the corresponding author, subject to compliance with the legal authorization of the FCDS and the Florida Department of Health.
