# Supplementary material for: Racial/ethnic disparities in curative‐intent treatment for early‐stage non‐small cell lung cancer patients among heterogeneous Black populations: US‐born Black, Afro‐Haitian, West Indian Black, and Hispanic Black
Source: Cancer Med. 2024 Oct 8;13(19):e7449. doi: 10.1002/cam4.7449 (PMC11459681; doi:10.1002/cam4.7449)
Supplement: Supplementary file 1 — Data S1. [file CAM4-13-e7449-s002.docx]

Supplementary Figure 1. Flow diagram for patient selection and exclusion, Florida, 2005-2017

Patients diagnosed with early-stage NSCLC (AJCC stages I/II and/or SEER localized stage), including non-Hispanic Whites (NHW) and all Blacks (Non-Hispanic and Hispanic) born in other countries, from 2005 to 2017 (n=56,077)

Patients diagnosed with early-stage NSCLC (AJCC stages I/II and/or SEER localized stage) from 2005 to 2017 (n=55,993)

Excluded

- Cases diagnosed at autopsy (n=10)
- Patients who died prior to recommended surgery (n=60)
- Patients who died before recommended SBRT (n=120)
- Patients who refused both recommended surgery and radiation therapy (n=148)

Analysis population (n=55,655)

Excluded

- NHB born in counties other than US, Haiti, and West Indies (n=84)

| Supplemental Table 1. Full list of determinants of receipt of any curative-intent treatment (surgery and/or SBRT), Florida, 2005-2017 (N=55,655) | | | |
| --- | --- | --- | --- |
|  | Odds Ratio (95% CI) | | |
|  | Curative-intent treatment (surgery and/or SBRT) (*N*=39,566) vs. No curative-intent treatment (*N*=16,089) | | |
|  | Model 1 ^1^ | Model 2 ^2^ | Model 3 ^3^ |
| Age group |  |  |  |
| <55 | 1 [Reference] | 1 [Reference] | 1 [Reference] |
| 55-64 | 0.89 (0.81 to 0.98) | 0.96 (0.87 to 1.05) | 0.84 (0.76 to 0.94) |
| 65-74 | 0.87 (0.80 to 0.95) | 0.97 (0.89 to 1.06) | 0.74 (0.67 to 0.83) |
| 75-84 | 0.61 (0.56 to 0.66) | 0.69 (0.63 to 0.76) | 0.51 (0.46 to 0.57) |
| ≥85 | 0.25 (0.23 to 0.28) | 0.28 (0.25 to 0.31) | 0.22 (0.19 to 0.25) |
| Race/ethnicity |  |  |  |
| White | 1 [Reference] | 1 [Reference] | 1 [Reference] |
| Total Black^4^ | 0.52 (0.49 to 0.55) | 0.52 (0.49 to 0.56) | 0.65 (0.59 to 0.70) |
| U.S. born Black | 0.50 (0.47 to 0.54) | 0.50 (0.47 to 0.54) | 0.64 (0.59 to 0.69) |
| Afro-Haitians Black | 0.60 (0.45 to 0.82) | 0.57 (0.41 to 0.79) | 0.61 (0.41 to 0.89) |
| West Indian Black | 0.71 (0.56 to 0.91) | 0.79 (0.61 to 1.03) | 0.76 (0.56 to 1.02) |
| Hispanic Black | 0.61 (0.44 to 0.84) | 0.58 (0.41 to 0.81) | 0.57 (0.39 to 0.82) |
| CCI |  |  |  |
| CCI=0 | - | 1 [Reference] | 1 [Reference] |
| 1 ≤ CCI ≤ 2 | - | 0.70 (0.66 to 0.73) | 0.85 (0.80 to 0.89) |
| CCI ≥ 3 | - | 0.53 (0.50 to 0.56) | 0.68 (0.63 to 0.73) |
| Sex |  |  |  |
| Male | - | - | 1 [Reference] |
| Female | - | - | 1.21 (1.15 to 1.26) |
| Smoking status |  |  |  |
| Never Smoker | - | - | 1 [Reference] |
| Current smoker | - | - | 0.92 (0.85 to 1.00) |
| Former smoker | - | - | 1.15 (1.06 to 1.25) |
| Unknown | - | - | 0.88 (0.80 to 0.96) |
| Poverty level |  |  |  |
| 0% – <5% poverty | - | - | 1 [Reference] |
| 5% – <10% poverty | - | - | 0.91 (0.84 to 0.99) |
| 10% – <20% poverty | - | - | 0.79 (0.73 to 0.85) |
| 20% – 100% poverty | - | - | 0.68 (0.62 to 0.74) |
| Unknown | - | - | 0.90 (0.70 to 1.16) |
| Health insurance status |  |  |  |
| Private | - | - | 1 [Reference] |
| Not Insured | - | - | 0.48 (0.38 to 0.59) |
| Self-Pay | - | - | 0.43 (0.35 to 0.53) |
| Medicaid | - | - | 0.56 (0.52 to 0.61) |
| Medicare | - | - | 0.84 (0.80 to 0.89) |
| Other government insurances | - | - | 0.78 (0.68 to 0.89) |
| Unknown | - | - | 0.61 (0.52 to 0.73) |
| Region |  |  |  |
| South Florida | - | - | 1 [Reference] |
| Tampa Bay | - | - | 0.77 (0.71 to 0.82) |
| Central Florida | - | - | 0.76 (0.70 to 0.81) |
| Northeast Florida | - | - | 0.70 (0.64 to 0.75) |
| Southwest Florida | - | - | 0.82 (0.75 to 0.88) |
| Northwest Rural | - | - | 0.70 (0.64 to 0.77) |
| Marital status |  |  |  |
| Not married | - | - | 1 [Reference] |
| Married | - | - | 1.35 (1.29 to 1.42) |
| Unknown | - | - | 1.11 (0.96 to 1.28) |
| Diagnosis Year |  |  |  |
| 2005-2009 | - | - | 1 [Reference] |
| 2010-2014 | - | - | 1.71 (1.62 to 1.81) |
| 2015-2017 | - | - | 1.71 (1.60 to 1.82) |
| AJCC stage |  |  |  |
| Stage I | - | - | 1 [Reference] |
| Stage II | - | - | 0.58 (0.54 to 0.61) |
| Unknown | - | - | 0.29 (0.27 to 0.32) |
| Histology |  |  |  |
| Adenocarcinoma | - | - | 1 [Reference] |
| Squamous cell carcinoma | - | - | 0.56 (0.53 to 0.59) |
| Large cell carcinoma | - | - | 0.58 (0.50 to 0.67) |
| Unspecified | - | - | 0.06 (0.05 to 0.07) |
| NSCLC NOS | - | - | 0.32 (0.30 to 0.35) |
| Cancer sequence number |  |  |  |
| 1 primary in the patient’s lifetime | - | - | 1 [Reference] |
| 1^st^ of 2 or more primaries | - | - | 2.13 (1.97 to 2.31) |
| 2nd of 2 or more primaries | - | - | 1.27 (1.20 to 1.34) |
| ≥3^rd^ of 2 or more primaries | - | - | 1.50 (1.37 to 1.63) |
| Abbreviations: AJCC, American Joint Committee on Cancer; CCI, Charlson Comorbidity Index; NSCLC, non-small cell lung cancer; NOS, not otherwise specified; SBRT, stereotactic body radiation therapy.  ^1^ Model was adjusted for age.  ^2^ Model was adjusted for age and comorbidities.  ^3^ Model was adjusted for sociodemographic (age, sex, poverty level, health insurance status, region, marital status), smoking status and clinical factors (diagnosis year, AJCC stage, histology, cancer sequence number and comorbidities).  ^4^ ORs for total Black were obtained from the model combined all patients from Black ethnicity groups. | | | |
